# Supplementary material for: A single cell genomics atlas of the Drosophila larval eye reveals distinct photoreceptor developmental timelines
Source: Nat Commun. 2023 Nov 8;14:7205. doi: 10.1038/s41467-023-43037-0 (PMC10632452; doi:10.1038/s41467-023-43037-0)
Supplement: Supplementary file 1 — Supplementary Information [file 41467_2023_43037_MOESM1_ESM.pdf]

Supplementary Note 1

Features of the putative Late R cell cluster

Our cluster plots show a group of cells that we refer to as a putative Late R cell cluster (Figs. 1D and Supplementary Fig. 6A). The number of features and counts per cell is lower in the Late R cell cluster compared to other cell clusters. We currently do not know the origin of the cells in this cluster and future work is required to confirm the identity of these cells. However, we speculate that this cluster may represent R cell strands that merge into a single Late R cell cluster as they mature. We also observe R cell strands and the Late R cell cluster when we tested several different UMAP hyperparameters and random seeds (Supplementary Fig. 8A-I). Moreover, expression of most R cell subtype-specific genes (e.g., *sens*, *ro* and *svp*) decreases in the Late R cell cluster, which instead expresses many markers related to axogenesis, axon-pathfinding and synapse formation (Supplementary Fig. 6F). Differential gene expression analyses identified several Late R cell cluster marker genes (Supplementary Fig. 6F and Supplementary Data 1). Since our scRNA data shows mature cells on the right of the cluster plot, we expect Late R cells to be present in the posterior-most columns of the eye disc. For example, *onecut*, *chaoptin* (*chp*) and *quiver* (*qvr*), show expression in the far right of the cluster plot, including the Late R cell cluster (Supplementary Fig. 6G,I,K). We determined the *in vivo* expression patterns of these genes by driving *UAS-mCherry-nls* using either *onecut*-, *chp*- or *qvr-T2A-Gal4* constructs and costained eye discs with mCherry and Elav (a pan-neuronal marker). Consistent with the expression patterns in our scRNA-seq data, we observe mCherry only in posterior R cell columns and this costains with Elav for all three *T2A-Gal4* lines (Supplementary Fig. 6H-H'', 6J-J' and 6L-L''). Moreover, expression of these genes is progressively restricted from the posterior/right end of the R cell strands to the distal tip of the Convergence cluster. Specifically, *onecut* is expressed in distal R cell strands and the Late R cell cluster, *chp* is expressed only in the Late R cell cluster but not in R cell strands, and *qvr* is expressed only on the posterior/right side of the Late R cell cluster. Again, the *in vivo* mCherry

patterns resemble the expression patterns observed in our data, with progressive restriction of expression to more posterior columns of the eye disc (Supplementary Fig. 6G,I,K). These data suggest that our data provides an accurate two-dimensional representation of the *in vivo* expression patterns of genes in the Late R cell cluster (and distal tips of the R7 and R8 strands).

To determine whether the Late R cell cluster consists of highly-related, but distinguishable multimodal cell types or if the cluster consists of mature R cells that have assumed a common transcriptional identity, we subclustered the Convergence cluster and performed PCA. PC1 captured 59% of the total variation (Supplementary Data 5) in the Late R cell cluster, suggesting that the genes along this principal component vary most and represent the identity of this cluster (Supplementary Data 4). We observe that many of the top PC1 genes are involved in axogenesis and synapse function, with expression in distal tips of R cell strands and the Late R cell cluster. This may be expected because posterior R cells are actively projecting axons and preparing for synapsis in the brain at this developmental time point. This suggests that the Late R cell cluster consists of R cell subtypes whose transcriptomes are dominated by high expression of axon-related genes. Therefore, cell type-specific gene expression is less prominent and the R cell strands merge into a common cluster. However, individual R cell subtype identities may persist in the Late R cell cluster.

A. Sequencing Metrics	
Total cell number	26,999
Means reads per cell	76,014
Number of reads	2.1 billion
Median genes per cell	2,173
Fraction reads in cells	94%
Median UMI counts per cell	10,089

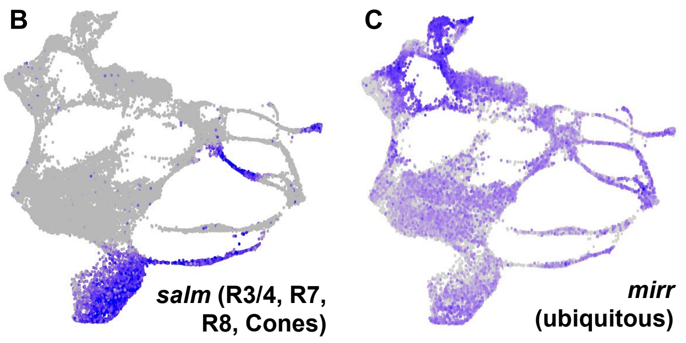

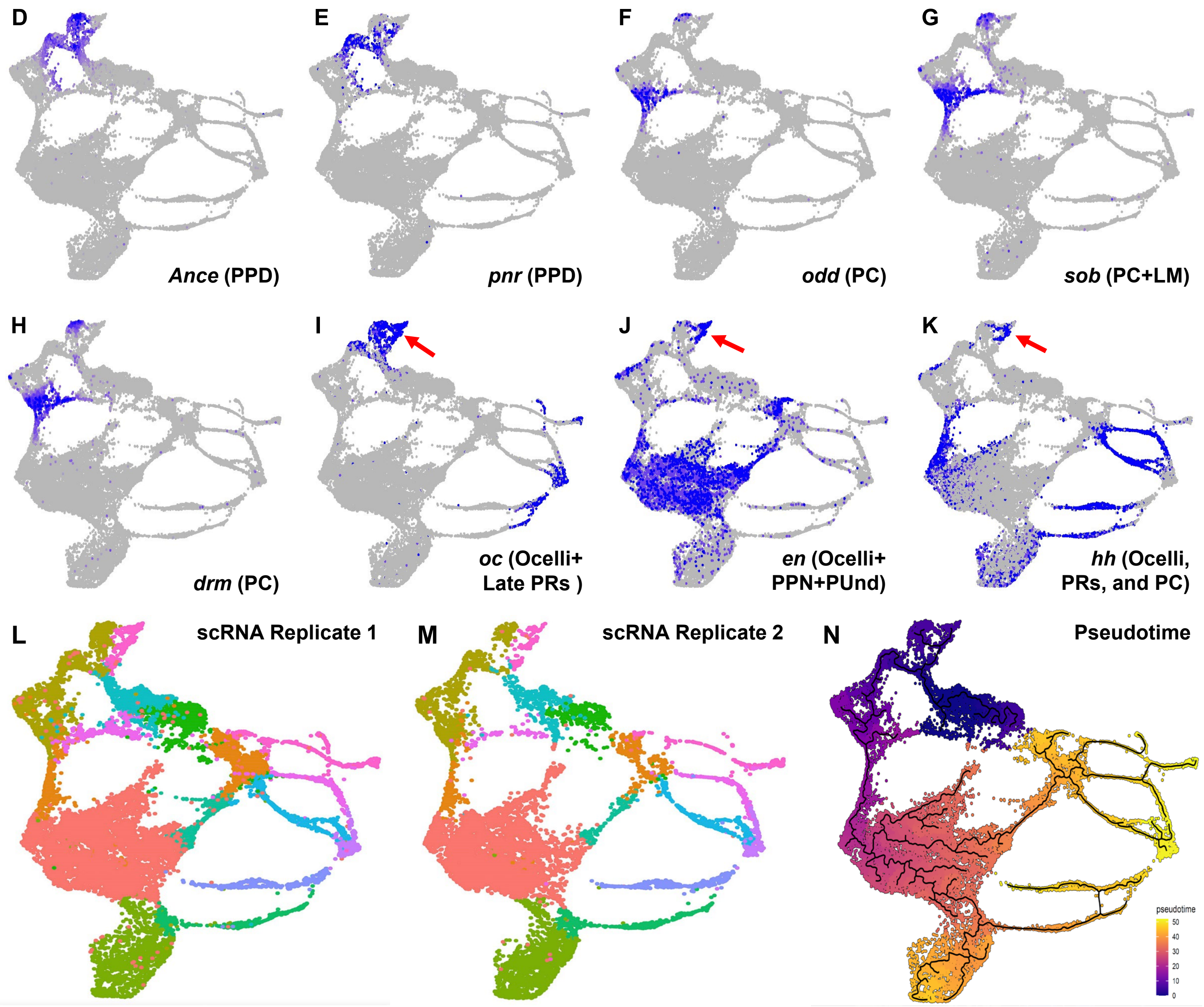

Supplementary Fig. 1: scRNA-seq metrics and identification of the PPD and Oc cell clusters in scRNA-seq data. A. Table showing quality metrics of the larval eye scRNA-seq data set. **B.** FeaturePlot showing *salm* expression in R3/4, R7, cones and late R8. The *salm* FeaturePlot expression is consistent with published studies. **C.** FeaturePlot of *mirr* showing ubiquitous expression without an apparent dorsal cluster. **D-K.** FeaturePlots of known marker genes used to identify the PPD, PC and OC clusters. **D.** FeaturePlot of the PPD marker *Angiotensin converting enzyme* (*Ance*) shows expression in the PPD. **E.** FeaturePlot for *pannier* (*pnr*) in the PPD cell cluster. **F-H.** FeaturePlots for *odd skipped* genes. *odd skipped* (*odd*, **F**), *sister of odd and bowl* (*sob*, **G**), and *drumstick* (*drm*, **H**) show expression specifically in the PC cell cluster. **I-K**. FeaturePlots showing expression of ocelli markers (red arrows): *ocelliless* (*oc*, **I**), *engrailed* (*en*, **J**), and *hedgehog* (*hh*, **K**). **L,M**. UMAP plots showing two biological replicates of scRNA-seq from late larval eye discs. Each cell type is well represented in each biological replicate. **N.** UMAP cluster plot showing pseudotime analysis of scRNA-seq data. Dark blue indicates early and yellow denotes late pseudotime. The UMAP plot shows the expected pseudotime profile with AUnd cells showing early pseudotime while the Late R cell cluster is late.

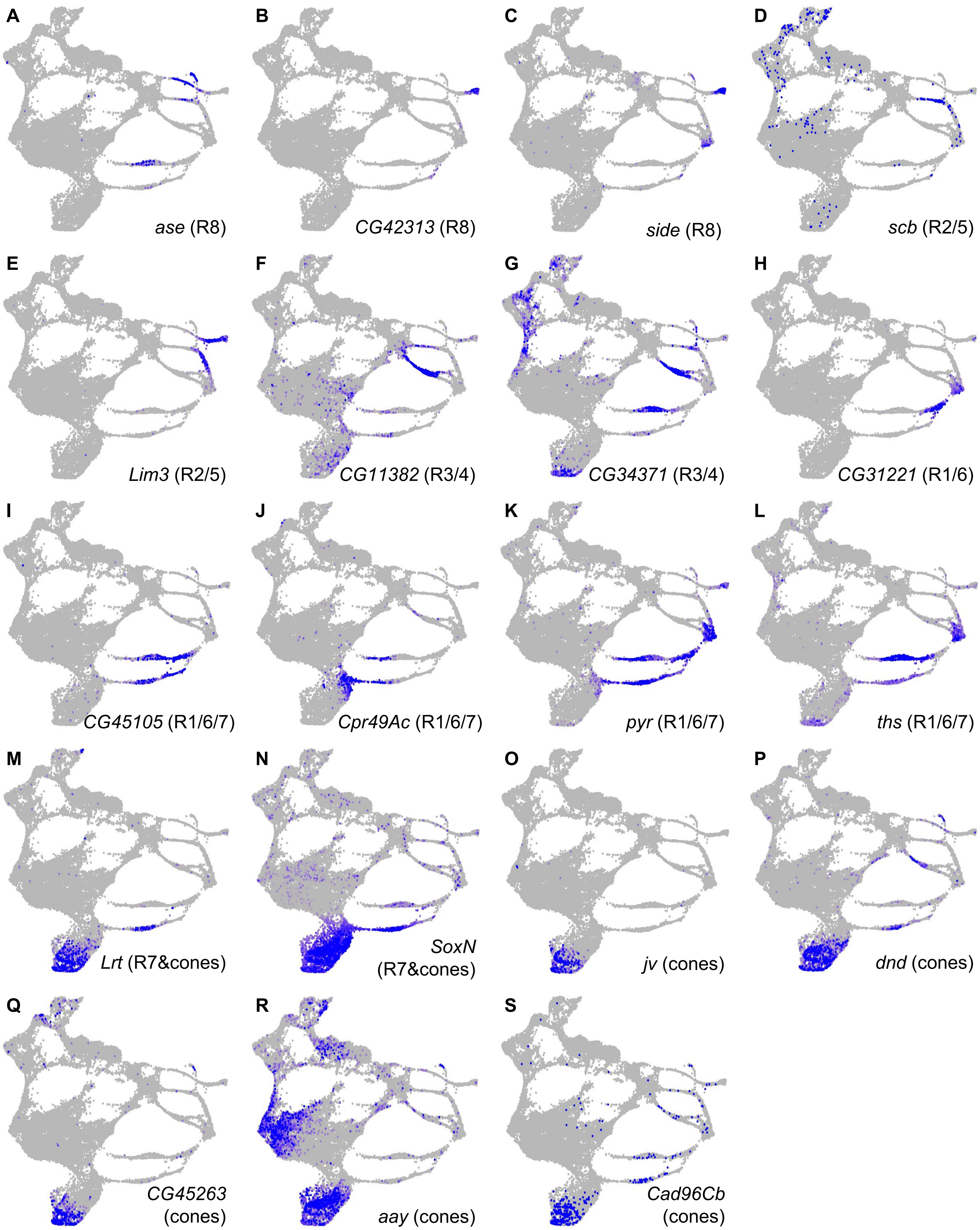

A
ase (R8)
B
CG42313 (R8)
C
side (R8)
D
scb (R2/5)
E
Lim3 (R2/5)
F
CG11382 (R3/4)
G
CG34371 (R3/4)
H
CG31221 (R1/6)
I
CG45105 (R1/6/7)
J
Cpr49Ac (R1/6/7)
K
pyr (R1/6/7)
L
ths (R1/6/7)
M
Lrt (R7&cones)
N
SoxN (R7&cones)
O
jv (cones)
P
dnd (cones)
Q
CG45263 (cones)
R
aay (cones)
S
Cad96Cb (cones)

Supplementary Fig. 2: Identification of novel cell type-specific markers from scRNA-seq data. A-S. FeaturePlots showing the expression and distribution of novel R cell and cone cell cluster markers. *asense* (*ase*, **A**) and *CG42313* (**B**) are expressed in R8. **C.** *sidestep* (*side*) is predominantly expressed in late R8. *scab* (*scb*, **D**) and *Lim3* (**E**) show expression in the R2/5 cluster. *Lim3* is also expressed in R8. FeaturePlots of *CG11382* (**F**) and *CG34371* (**G**) show mRNA in R3/4. *CG34371* is also expressed in R1/6. **H.** FeaturePlot of *CG31221* showing expression in late R1/6 and the Late R cell cluster. **I-L**. FeaturePlots of genes showing R1/6- and R7-specific expression: *CG45105* (**I**), *Cuticular protein 49Ac* (*Cpr49Ac*, **J**), *pyramus* (*pyr*, **K**), and *thisbe* (*ths*, **L**)*. pyr* and *ths* also show expression in the Late R cell cluster. *Leucine-rich tendon-specific protein* (*Lrt*, **M**) and *SoxN* (**N**) are expressed in the R7 and cone cell clusters. **O-S.** FeaturePlots showing the expression of novel cone cell markers: *javelin* (*jv*, **O**), *dead end* (*dnd*, **P**), *CG45263* (**Q**), *astray* (*aay*, **R**), and *Cadherin 96Cb* (*Cad96Cb*, **S**). *aay* is also expressed in the PUnd cell cluster while *dnd* and *Cad96Cb* also show sporadic and weak expression in some R cells.

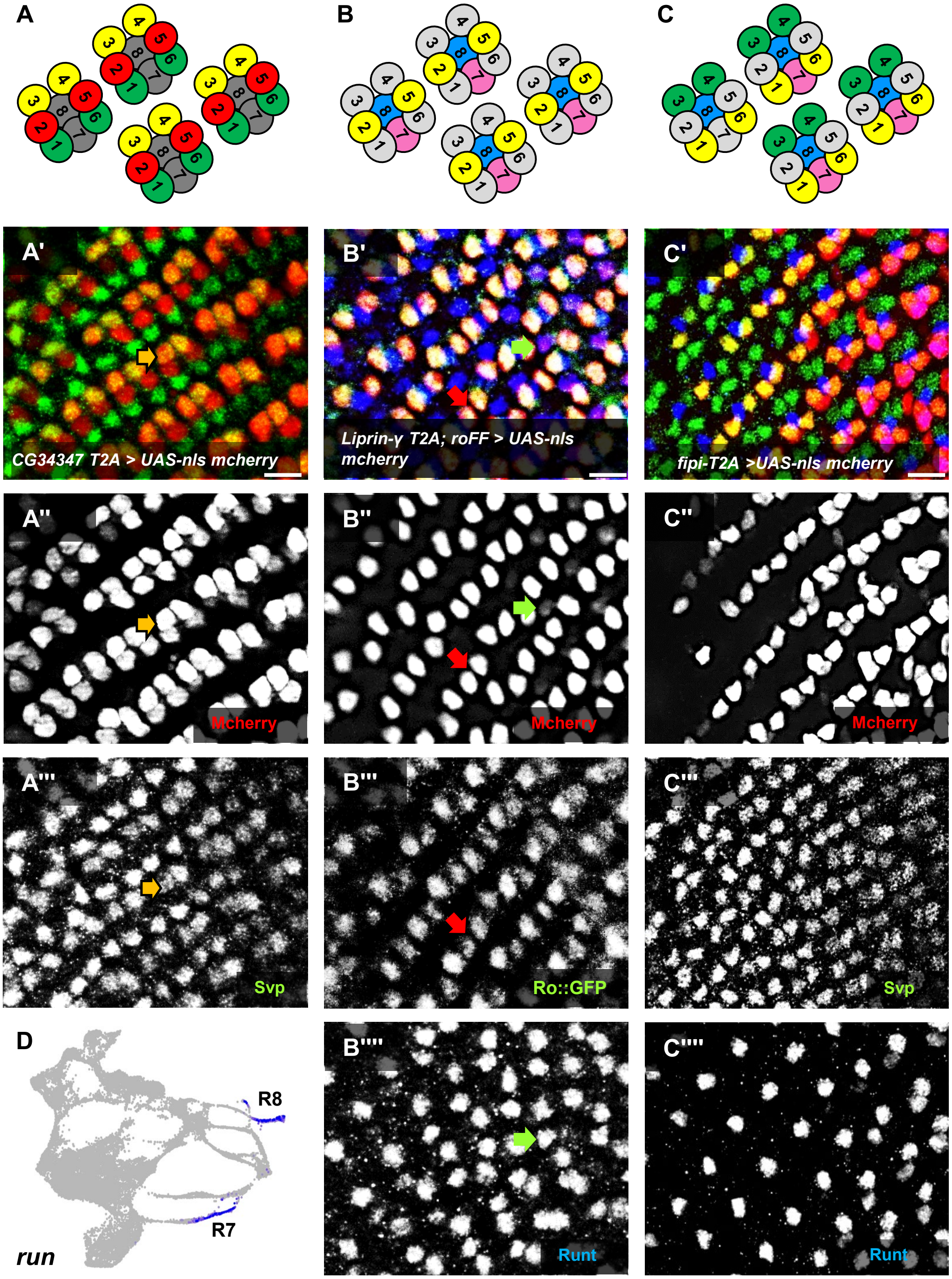
A
B
C
A'
CG34347 T2A > UAS-nls mcherry
B'
Liprin-γ T2A; roFF > UAS-nls mcherry
C'
fipi-T2A >UAS-nls mcherry
A''
Mcherry
B''
Mcherry
C''
Mcherry
A'''
Svp
B'''
Ro::GFP
C'''
Svp
D
R8
R7
run
B''''
Runt
C''''
Runt

Supplementary Fig. 3: Identification and *in vivo* validation of novel markers.

A-C. Schematics of four ommatidia with eight (R1-R8) photoreceptors each. The colors denote the expression observed in **A'-C'. A'-A'''.** *CG34347-T2A-Gal4>UAS-nls-mCherry* eye disc colabeled with mCherry (red) and Svp (green). Two Svp-positive cells also costain for mCherry (orange arrow in **A',A''** and **A'''**). **B'-B''''.** Colabeling of a *liprin-γ-T2A-Gal4; ro-flipflop-GFP>UAS-nls-mCherry* larval eye disc with mCherry (red), Run (blue) and GFP (green). *liprin-γ-T2A-Gal4* drives mCherry expression in R2/5 (red arrow in **B'-B'''**) and faintly in R7 (green arrow in **B'**, **B''** and **B'''**), consistent with the expression observed in the FeaturePlot (Figure 3C). **C'-C'''.** Costaining of a *fipi-T2A-Gal4>UAS-nls-mCherry* eye disc with mCherry (red), Svp (green) and Runt (blue). Two mCherry-positive cells costain with Svp (purple arrow in **C'-C'''**). A third cell costains with Run (yellow arrow in **C'**,**C''** and **C''''**). **D.** FeaturePlot for *run* shows expression in late R7 and R8. All scale bars: 20 µm

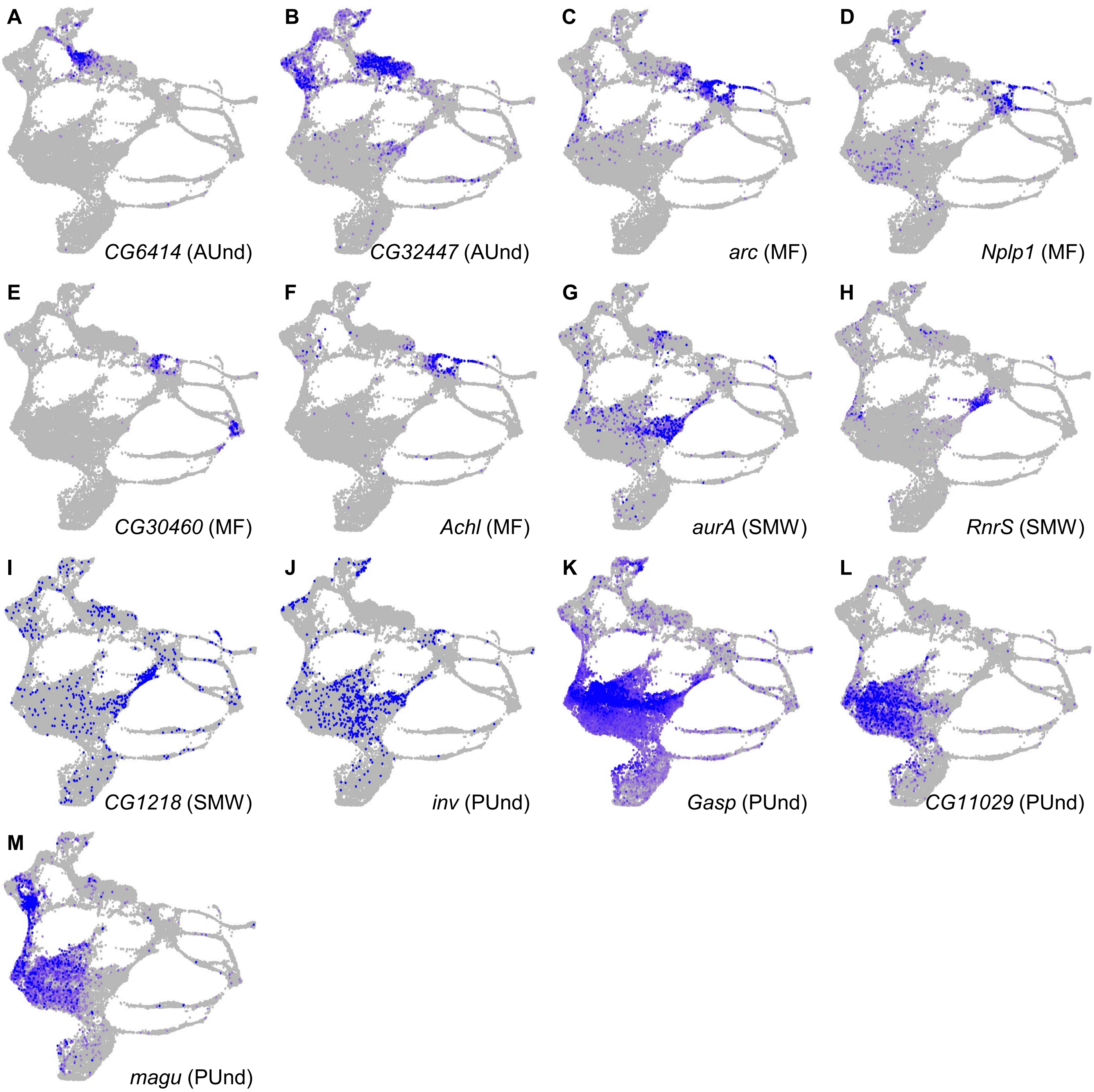

A
CG6414 (AUnd)
B
CG32447 (AUnd)
C
arc (MF)
D
Nplp1 (MF)
E
CG30460 (MF)
F
Achl (MF)
G
aurA (SMW)
H
RnrS (SMW)
I
CG1218 (SMW)
J
inv (PUnd)
K
Gasp (PUnd)
L
CG11029 (PUnd)
M
magu (PUnd)

Supplementary Fig. 4: Novel markers of undifferentiated cell clusters. *CG6414* (**A**), and *CG32447* (**B**) are novel AUnd markers. *arc* (**C**), *Neuropeptide-like precursor 1* (*Nplp1*, **D**), *CG30460* (**E**), and *Achilles* (*Achl*, **F**) are primarily expressed in the MF. *aurora A* (*aurA*, **G**), *Ribonucleoside diphosphate reductase small subunit* (*RnrS*, **H**), and *CG1218* (**I**) are three novel SMW markers. **K-N.** Novel PUnd markers: *invected* (*inv*, **J**), *Gasp* (**K**), *CG11029* (**L**), and *magu* (**M**).

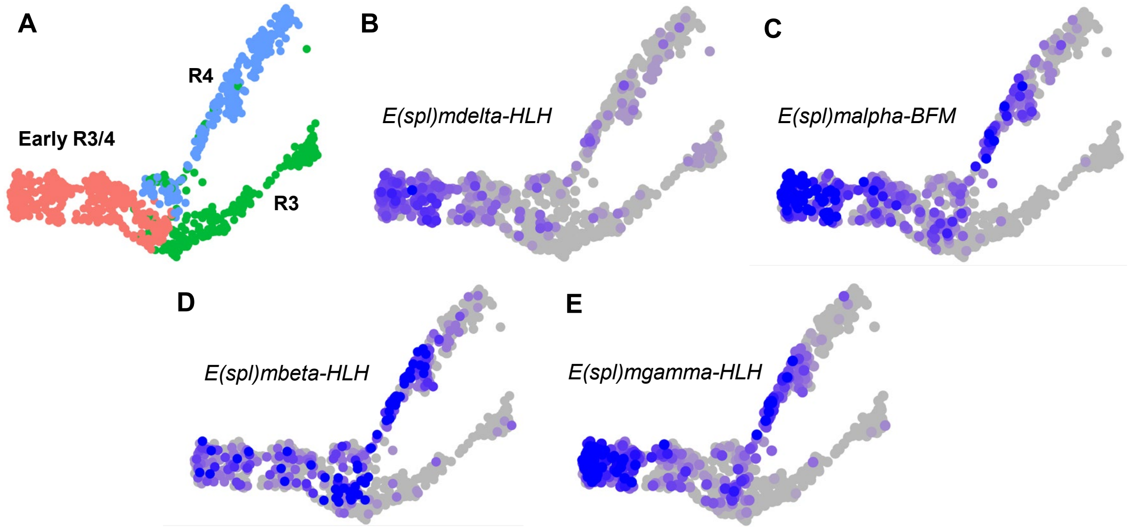
A
R4
Early R3/4
R3
B
E(spl)mdelta-HLH
C
E(spl)malpha-BFM
D
E(spl)mbeta-HLH
E
E(spl)mgamma-HLH

Supplementary Fig. 5: The R4 cell cluster shows higher levels of *Enhancer of split* gene expression. A. UMAP plot showing the split in the R3/4 strand. **B-E.** FeaturePlots showing the expression of several *E(spl)* genes: **B.** *E(spl)mdelta-HLH*, **C.** *E(spl)malpha-BFM*, **D.** *E(spl)mbeta-HLH*, and **E.** *E(spl)mgamma-HLH*. *E(spl)* genes show higher levels of expression in R4 compared to R3.

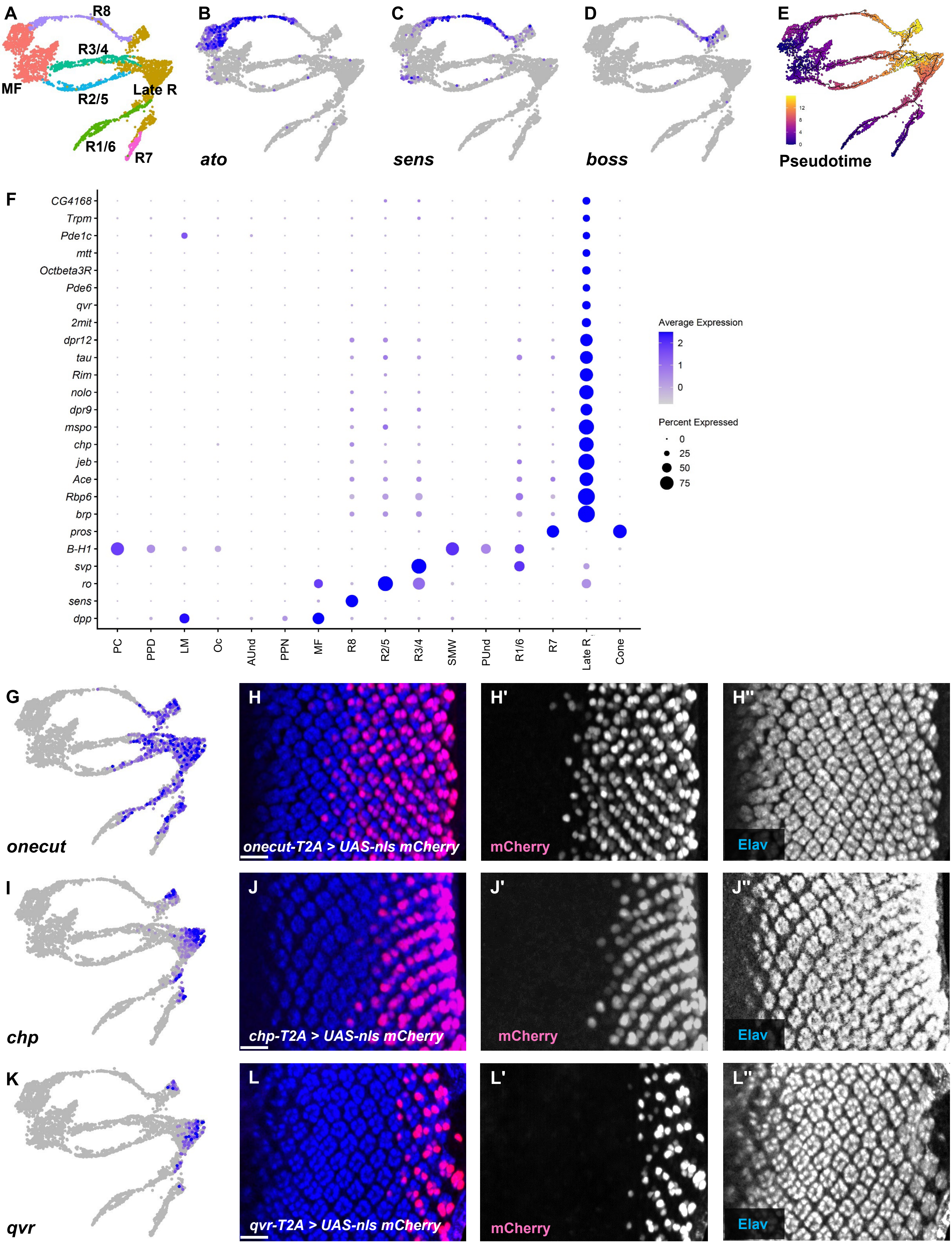
A
R8
R3/4
MF
R2/5
Late R
R1/6
R7
B
ato
C
sens
D
boss
E
Pseudotime
F
CG4168
Trpm
Pde1c
mtt
Octbeta3R
Pde6
qvr
2mit
dpr12
tau
Rim
nolo
dpr9
mspo
chp
jeb
Ace
Rbp6
brp
pros
B-H1
svp
ro
sens
dpp
PC
PPD
LM
Oc
AUnd
PPN
MF
R8
R2/5
R3/4
SMW
PUnd
R1/6
R7
Late R
Cone
Average Expression
2
1
0
Percent Expressed
0
25
50
75
G
onecut
H
onecut-T2A > UAS-nls mCherry
H'
mCherry
H''
Elav
I
chp
J
chp-T2A > UAS-nls mCherry
J'
mCherry
J''
Elav
K
qvr
L
qvr-T2A > UAS-nls mCherry
L'
mCherry
L''
Elav

Supplementary Fig. 6: Distinct strands of photoreceptor cells are connected to undifferentiated clusters and then merge into a single Late R cell cluster. A. UMAP cluster plot showing only the MF and R cells. The R cell clusters exhibit a spatiotemporal dimension with less mature R cells near the MF and more mature R cells near the Late R cell cluster. **B.** FeaturePlot showing the expression of *ato* in the MF and R8 near the MF*.* **C.** FeaturePlot showing *sens* expression distributed throughout most of the R8 strand. **D.** FeaturePlot showing *boss* expression in more mature R8 cells. **E.** Trajectory analysis of the MF and R cells showing early to late pseudotime along each R cell strand. Purple indicates early pseudotime and late pseudotime is denoted as yellow. **F.** DotPlot showing expression of R cell-, cone- and Late R cell-specific markers. In the Late R cell cluster, cell type-specific gene expression is less prominent. **G.** FeaturePlot showing *onecut*, which is expressed in distal tips of R cells and the Late R cell cluster. **H-H"**. *onecut-T2A-Gal4>UAS-nls-mCherry* larval eye disc costained with mCherry (red) and Elav (blue). mCherry expression begins six to seven columns of ommatidia posterior to the MF. **I.** FeaturePlot showing expression of *chaoptin* (*chp*) specifically in the Late R cell cluster; no expression is observed in R cell strands. **J-J".** Colabeling of *chp-T2A-Gal4>UAS-mCherry-nls* with mCherry (red) and Elav (blue). mCherry is detected in a few posterior columns of ommatidia, consistent with the *chp* FeaturePlot. **K.** FeaturePlot of *quiver* (*qvr*) shows expression in the posterior (right) half of the Late R cell cluster. **L-L"**. *qvr-T2A-Gal4>UAS-nls-mCherry* eye disc costained with mCherry (red) and Elav (blue). mCherry is detected in just four ommatidial columns at the posterior margin of the eye disc. All scale bars: 20 µm

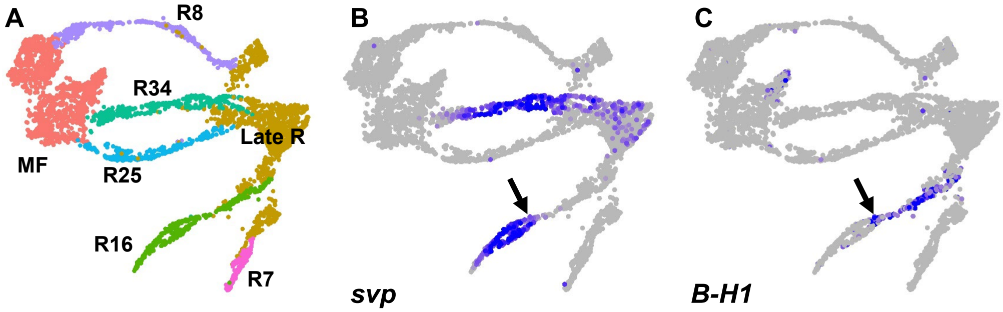
A
R8
R34
Late R
MF
R25
R16
R7
B
svp
C
B-H1

Supplementary Fig. 7: Photoreceptor strands comprise cells in a temporal developmental series.

A. UMAP plot showing the MF and R cells only. R cell subtype clusters appear as strands connected to the MF (R8, R2/5, and R3/4) or undifferentiated cells (R1/6 and R7). **B.** A FeaturePlot showing the expression of *svp* throughout the R3/4 strand but only in less mature R1/6 cells. **C.** *B-H1* FeaturePlot showing expression in mature but not developmentally younger R1/6 cells. *B-H1* mRNA is observed only after *svp* expression ceases along the strand.

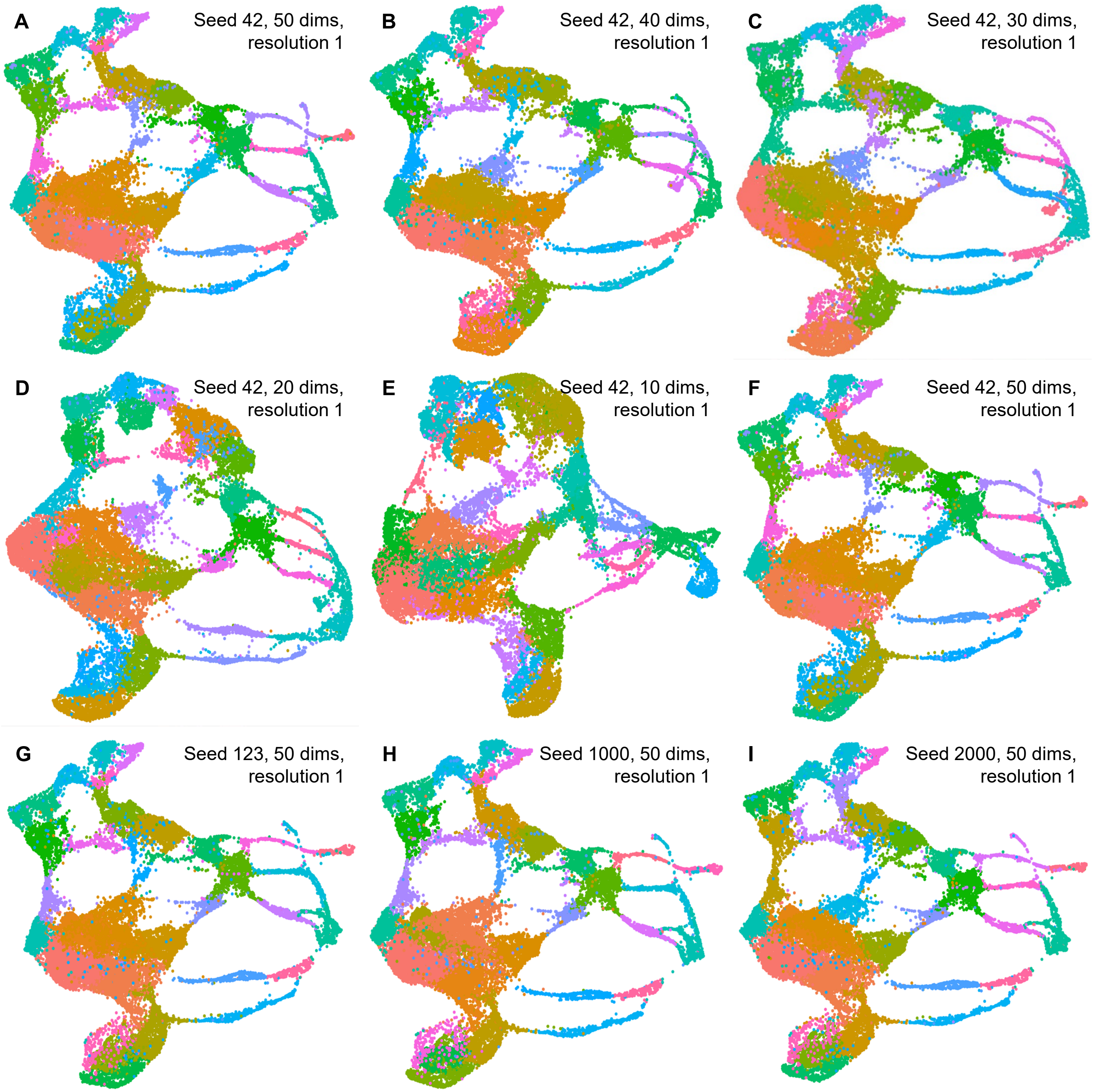

A
Seed 42, 50 dims, resolution 1
B
Seed 42, 40 dims, resolution 1
C
Seed 42, 30 dims, resolution 1
D
Seed 42, 20 dims, resolution 1
E
Seed 42, 10 dims, resolution 1
F
Seed 42, 50 dims, resolution 1
G
Seed 123, 50 dims, resolution 1
H
Seed 1000, 50 dims, resolution 1
I
Seed 2000, 50 dims, resolution 1

Supplementary Fig. 8: Altering parameters has only modest effects on scRNA-seq cluster plots. A-E. Cluster plots were generated with a random seed of 42 and a resolution of 1 but with different dimensions: 50 (**A**), 40 (**B**), 30 (**C**), 20 (**D**) and 10 (**E**). **F-I.** Cluster plots were generated with different random seeds: 42 (**F**) 123 (**G**) 1000 (**H**) and 2000 (**I**). All plots show photoreceptor strands that merge into a single cluster.

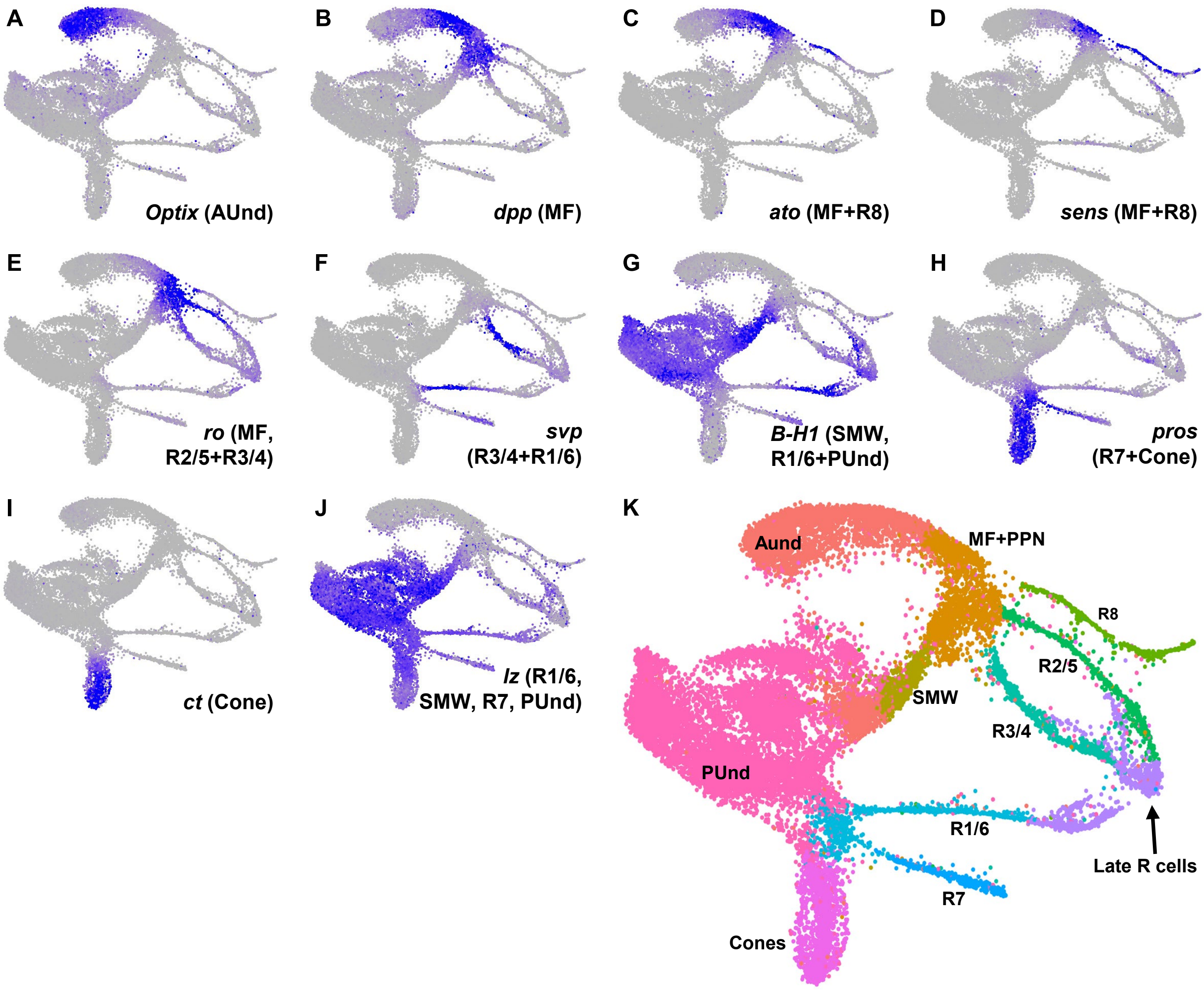
A
Optix (AUnd)
B
dpp (MF)
C
ato (MF+R8)
D
sens (MF+R8)
E
ro (MF, R2/5+R3/4)
F
svp (R3/4+R1/6)
G
B-H1 (SMW, R1/6+PUnd)
H
pros (R7+Cone)
I
ct (Cone)
J
lz (R1/6, SMW, R7, PUnd)
K
Aund
MF+PPN
R8
R2/5
SMW
R3/4
PUnd
R1/6
Late R cells
R7
Cones

Supplementary Fig. 9: Annotation of snATAC-seq cell clusters by imputation of RNA from scRNA-seq data. A-J. FeaturePlots showing the distribution of imputed RNA on snATAC-seq clusters. **A.** FeaturePlot showing *Optix* mRNA in the AUnd cluster. **B.** *decapentaplegic* (*dpp*) mRNA is predominantly detected in the MF. **C.** *atonal* (*ato*) mRNA is in the MF and R8 strand. **D.** *senseless* (*sens*) mRNA is specific to the MF and R8. **E.** *rough* (*ro*) mRNA is confined to the MF, R2/5 and R3/4 cell clusters. **F.** *seven up* (*svp*) mRNA is detected primarily in R3/4 and R1/6. **G.** *B-H1* mRNA is observed in R1/6 and PUnd. **H.** *prospero* (*pros*) is specifically present in the R7 and cone cell clusters. **I.** *cut* (*ct*) is completely confined to cone cells. **J.** FeaturePlot of *lozenge* (*lz*) showing expression primarily in R1/6, R7 and PUnd. **K.** The UMAP plot for late larval snATAC-seq data with cluster identities is shown for reference.

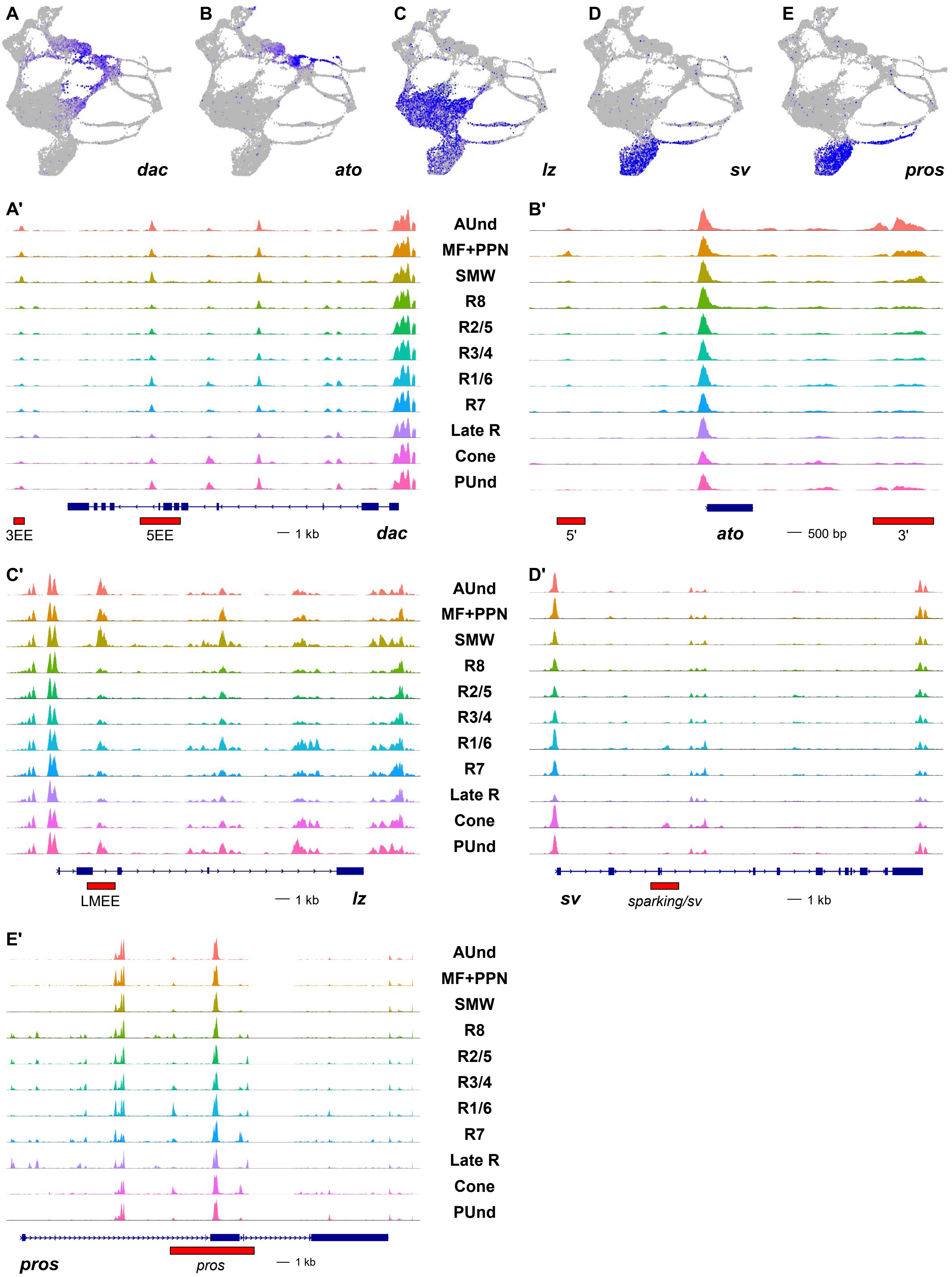
A
B
C
D
E
dac
ato
lz
sv
pros
A'
B'
AUnd
MF+PPN
SMW
R8
R2/5
R3/4
R1/6
R7
Late R
Cone
PUnd
3EE
5EE
1 kb
dac
5'
ato
500 bp
3'
C'
D'
LMEE
lz
sv
sparking/sv
E'
pros
pros

Supplementary Fig. 10: Validation of snATAC-seq clusters using known larval eye enhancers. A-E. FeaturePlots showing the expression of the known marker genes *dachshund* (*dac*, **A**), *ato* (**B**), *lz* (**C**), *shaven* (*sv*, **D**), and *pros* (**E**). **A'-E'** show snATAC-seq CoveragePlots of known enhancer regions corresponding to the genes shown in **A-E.** The red horizontal box in each CoveragePlot denotes the relative size and position of known enhancer sequences that are sufficient to drive reporter gene expression in the eye. **A'**. *dac* CoveragePlot showing the 3EE and 5EE enhancers. The peak corresponding to 3EE is specifically accessible in the AUnd, MF+PPN, and SMW cell clusters. **B'.** The 5' and 3' enhancers of *ato* span regions of accessible chromatin*.* **C'.** CoveragePlot of *lz* showing a cone-specific peak (albeit somewhat weak) in the same position as the LMEE enhancer. **D'**. CoveragePlot of *sv* showing a cone-specific peak. The *sparkling/sv* enhancer maps to this peak region. **E'**. *pros* CoveragePlot showing an R7-enriched peak that is within the *pros* enhancer.

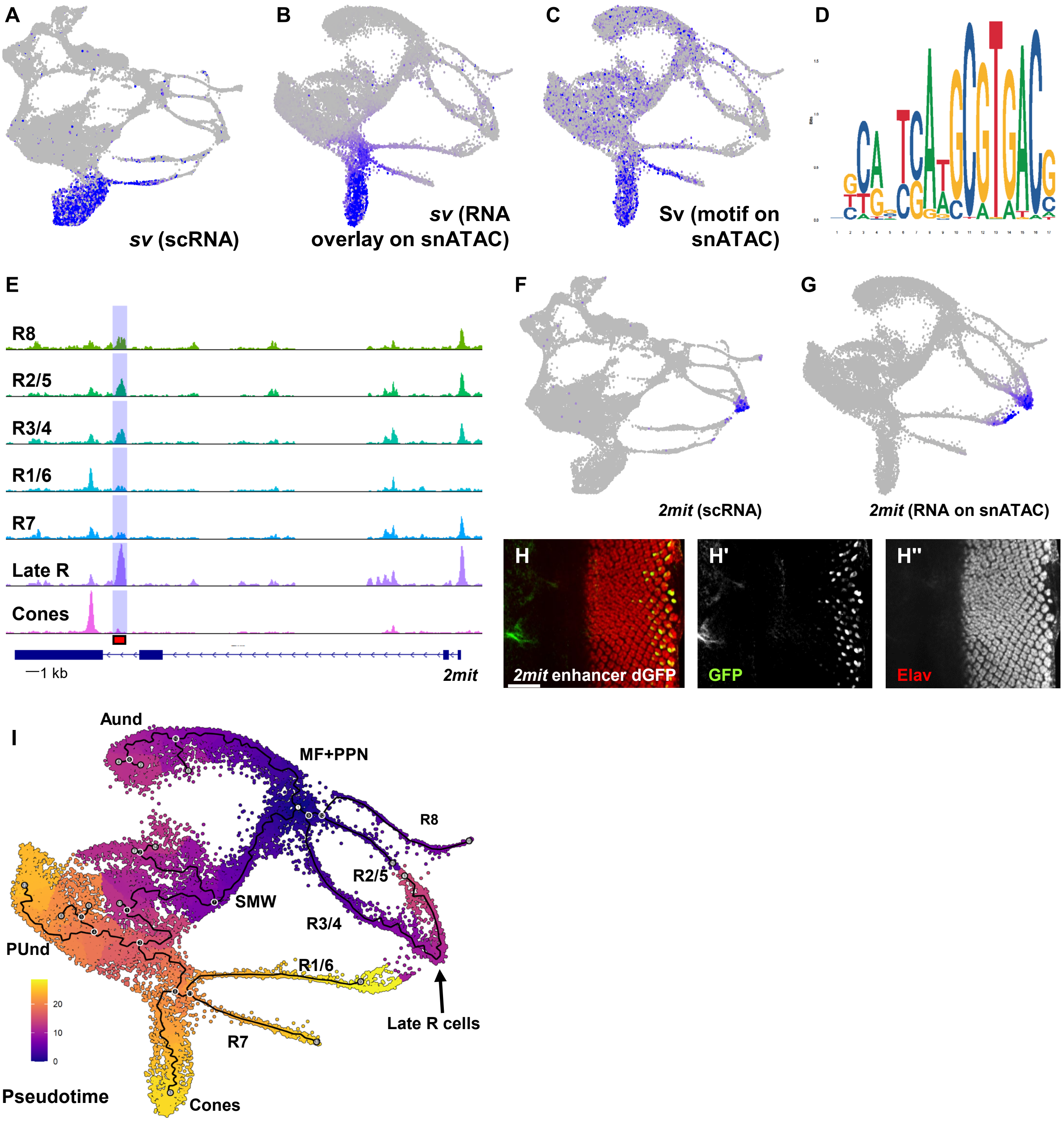
A
sv (scRNA)
B
sv (RNA overlay on snATAC)
C
Sv (motif on snATAC)
D
E
R8
R2/5
R3/4
R1/6
R7
Late R
Cones
1 kb
2mit
F
2mit (scRNA)
G
2mit (RNA on snATAC)
H
2mit enhancer dGFP
H'
GFP
H"
Elav
I
Aund
MF+PPN
R8
R2/5
SMW
R3/4
PUnd
R1/6
Late R cells
R7
Cones
20
10
0
Pseudotime

Supplementary Fig. 11: Validation of snATAC-seq clusters using motif analyses and identification of novel enhancers. A. scRNA-seq FeaturePlot of *sv* showing expression in R7 and cone cell clusters. **B.** FeaturePlot with the distribution of imputed *sv* RNA on snATAC-seq clusters showing RNA in R7 and cones. **C.** FeaturePlot showing the distribution of the Sv motif on the snATAC-seq UMAP plot. The Sv motif is enriched in R7 and cone cells. **D.** A Sv motif logo showing the Sv binding site. **E.** CoveragePlot of *2mit* showing a Late R cell-enriched peak highlighted in blue. The red bar indicates the DNA sequence used to make a reporter transgene. **F.** FeaturePlot of *2mit* showing expression mRNA expression in the Late R cell cluster. **G.** *2mit* RNA overlaid on the snATAC-seq UMAP plot showing Late R cell-specific distribution of mRNA. **H-H''.** Transgenic larval eye disc carrying a transgene with *2mit* peak-DNA driving destabilized GFP. The eye disc is costained with GFP (green) and Elav (red). GFP is detected in the most posterior ommatidial columns (**H** and **H'**). Scale bar: 20 μm **I.** snATAC-seq UMAP plot showing pseudotime trajectories inferred by Monocle 3. R1/6, R7 and cones show late pseudotime. The early pseudotime is indicated by purple, whereas yellow color represents a late pseudotime.

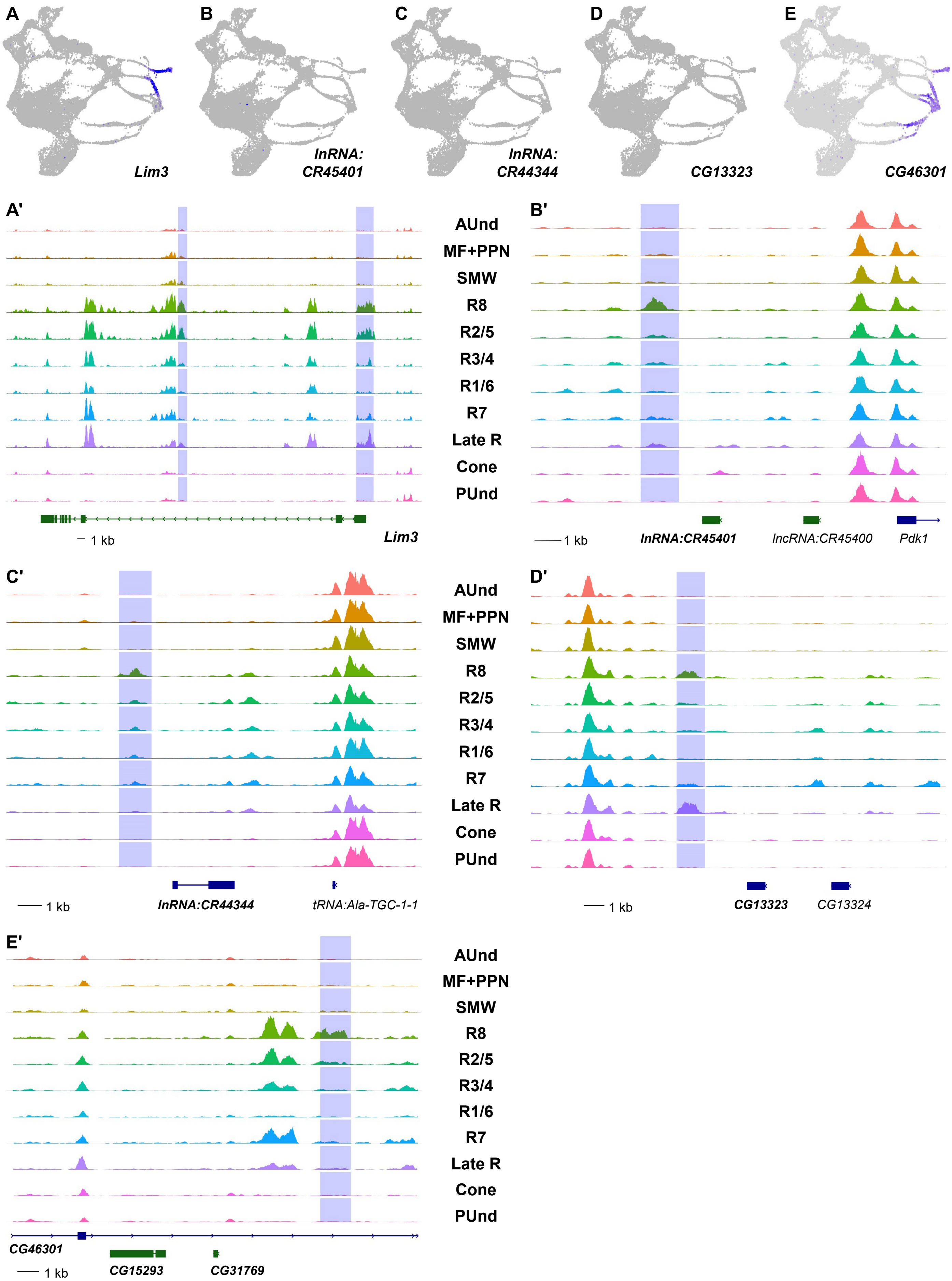
A
Lim3
B
lnRNA:
CR45401
C
lnRNA:
CR44344
D
CG13323
E
CG46301
A'
AUnd
MF+PPN
SMW
R8
R2/5
R3/4
R1/6
R7
Late R
Cone
PUnd
1 kb
Lim3
B'
1 kb
lnRNA:CR45401
lncRNA:CR45400
Pdk1
C'
1 kb
lnRNA:CR44344
tRNA:Ala-TGC-1-1
D'
1 kb
CG13323
CG13324
E'
CG46301
1 kb
CG15293
CG31769

Supplementary Fig. 12: Differentially accessible R8 cluster-specific snATAC-seq peaks.

A-E. FeaturePlots showing the expression of *Lim3* (**A**), *lnRNA:CR45401* (**B**), *lnRNA:CR44344* (**C**), *CG13323* (**D**), and *CG31769* (**E**). *Lim3* is expressed in R8 and R2/5. No mRNA is detected on the FeaturePlots of the other genes. **A'-E'.** CoveragePlots corresponding to the genes shown in **A-E.** All CoveragePlots show peaks that are primarily R8-specific.

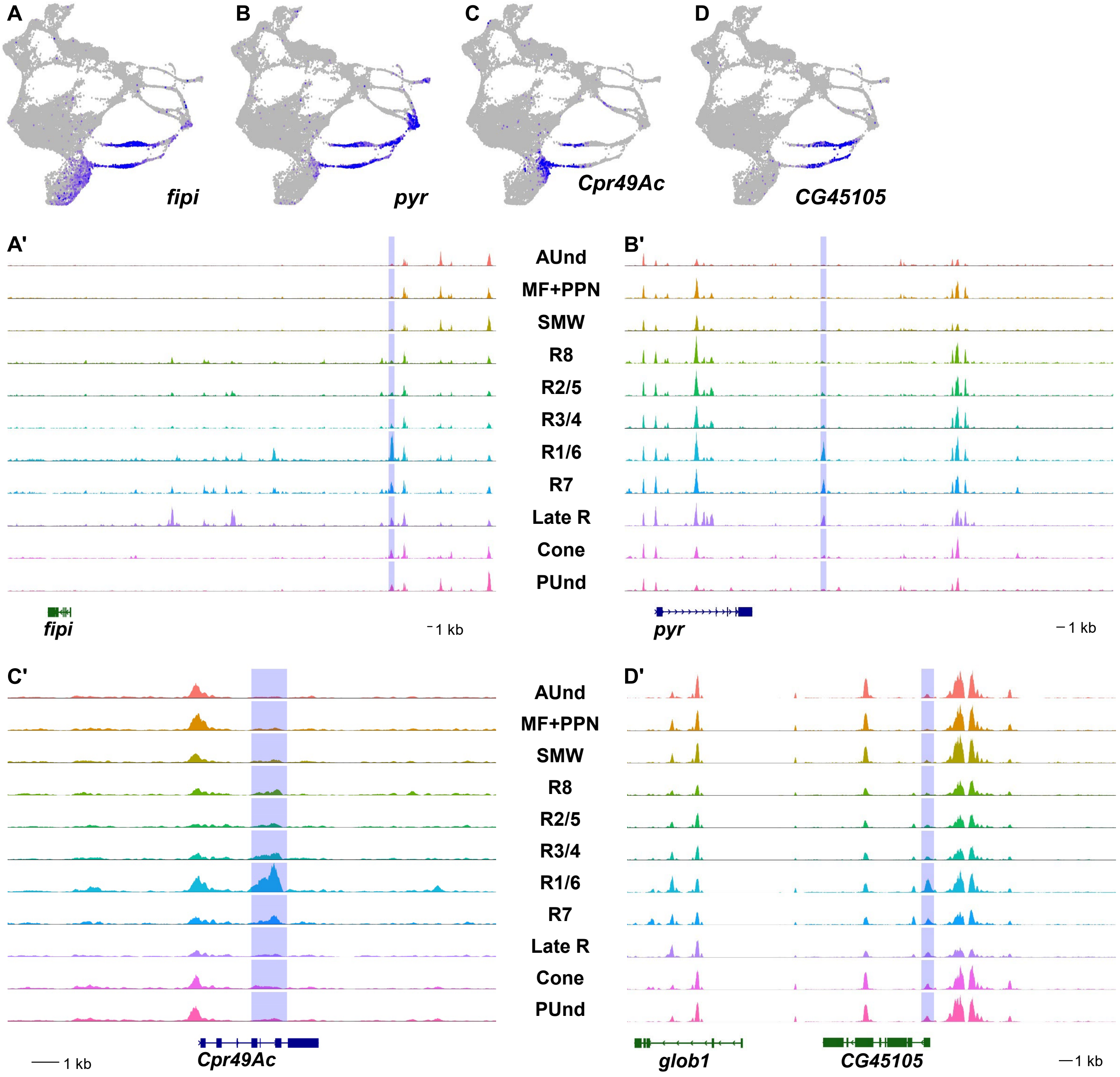

A
fipi
B
pyr
C
Cpr49Ac
D
CG45105
A'
B'
AUnd
MF+PPN
SMW
R8
R2/5
R3/4
R1/6
R7
Late R
Cone
PUnd
fipi
1 kb
pyr
1 kb
C'
D'
AUnd
MF+PPN
SMW
R8
R2/5
R3/4
R1/6
R7
Late R
Cone
PUnd
1 kb
Cpr49Ac
glob1
CG45105
1 kb

Supplementary Fig. 13: Differentially accessible R1/6 and R7 cluster-specific peaks. A-D. FeaturePlots showing the expression of *fipi* (**A**), *pyr* (**B**), *Cpr49Ac* (**C**), and *CG45105* (**D**). *fipi*, *pyr*, *Cpr49Ac*, and *CG45105* are expressed in both R1/6 and R7. *fipi* and *Cpr49Ac* are also expressed in the cone cell cluster while *pyr* is also present in the Late R cell cluster. **A'-D'.** CoveragePlots corresponding to the genes shown in **A-D**. All CoveragePlots show peaks largely specific to the R1/6 and R7 cell clusters.

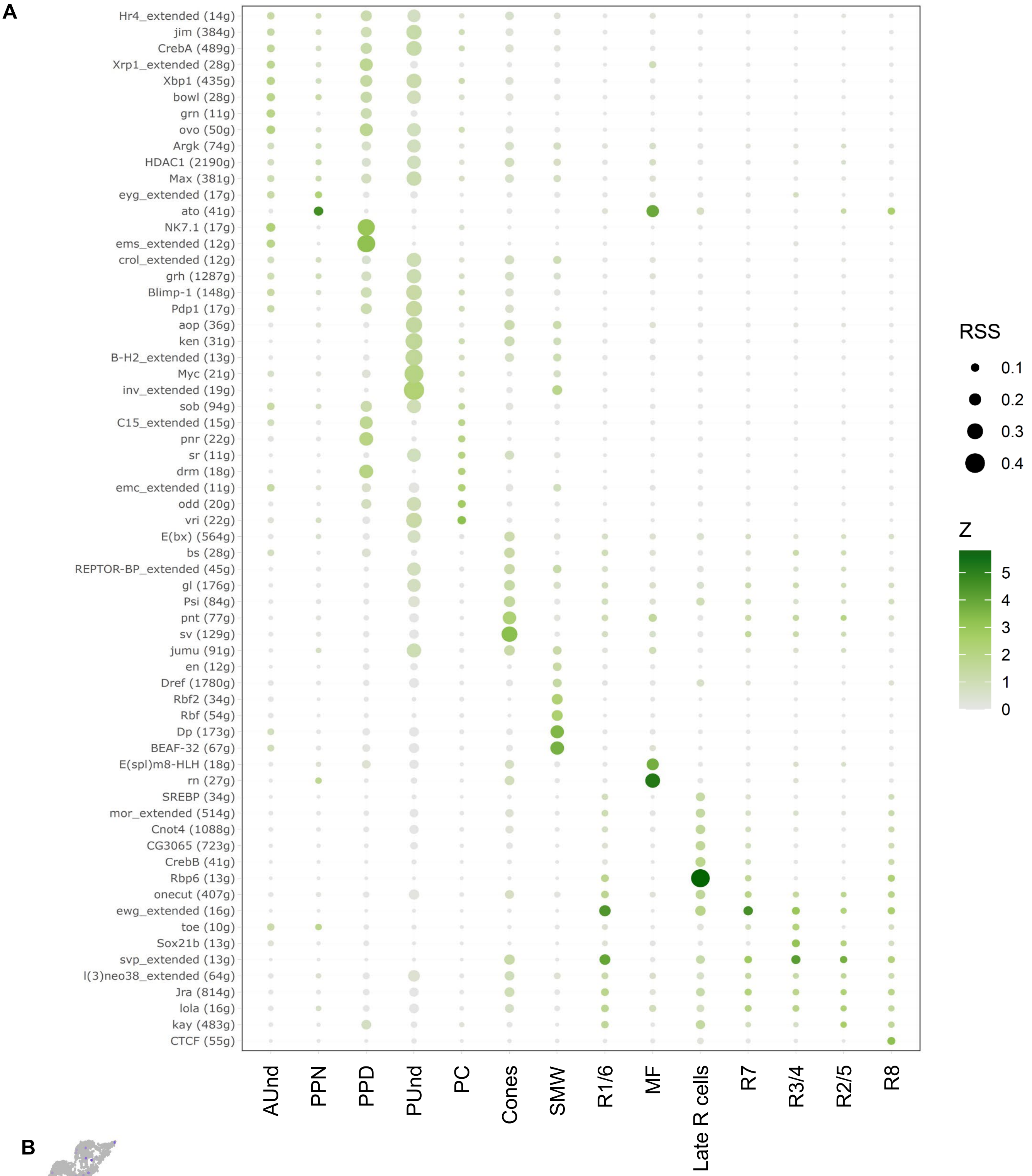
A
Hr4_extended (14g)
jim (384g)
CrebA (489g)
Xrp1_extended (28g)
Xbp1 (435g)
bowl (28g)
grn (11g)
ovo (50g)
Argk (74g)
HDAC1 (2190g)
Max (381g)
eyg_extended (17g)
ato (41g)
NK7.1 (17g)
ems_extended (12g)
crol_extended (12g)
grh (1287g)
Blimp-1 (148g)
Pdp1 (17g)
aop (36g)
ken (31g)
B-H2_extended (13g)
Myc (21g)
inv_extended (19g)
sob (94g)
C15_extended (15g)
pnr (22g)
sr (11g)
drm (18g)
emc_extended (11g)
odd (20g)
vri (22g)
E(bx) (564g)
bs (28g)
REPTOR-BP_extended (45g)
gl (176g)
Psi (84g)
pnt (77g)
sv (129g)
jumu (91g)
en (12g)
Dref (1780g)
Rbf2 (34g)
Rbf (54g)
Dp (173g)
BEAF-32 (67g)
E(spl)m8-HLH (18g)
rn (27g)
SREBP (34g)
mor_extended (514g)
Cnot4 (1088g)
CG3065 (723g)
CrebB (41g)
Rbp6 (13g)
onecut (407g)
ewg_extended (16g)
toe (10g)
Sox21b (13g)
svp_extended (13g)
l(3)neo38_extended (64g)
Jra (814g)
lola (16g)
kay (483g)
CTCF (55g)
AUnd
PPN
PPD
PUnd
PC
Cones
SMW
R1/6
MF
Late R cells
R7
R3/4
R2/5
R8
RSS
0.1
0.2
0.3
0.4
Z
5
4
3
2
1
0

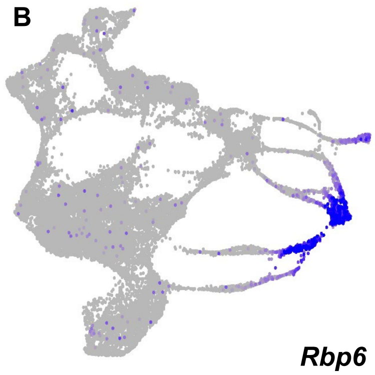
B
Rbp6

Supplementary Fig. 14: Top regulons of each scRNA-seq cell cluster identified using SCENIC. A. RSS plot showing the top regulons in each cell cluster. The color intensity of each dot is proportional to the expression level of each regulon. The size of the dot is proportional to the regulon specificity score. **B.** FeaturePlot showing the expression of *Rbp6* in the Late R cell cluster. *Rbp6* is highly expressed and specific to the Late R cell cluster and late R1/6 cells.

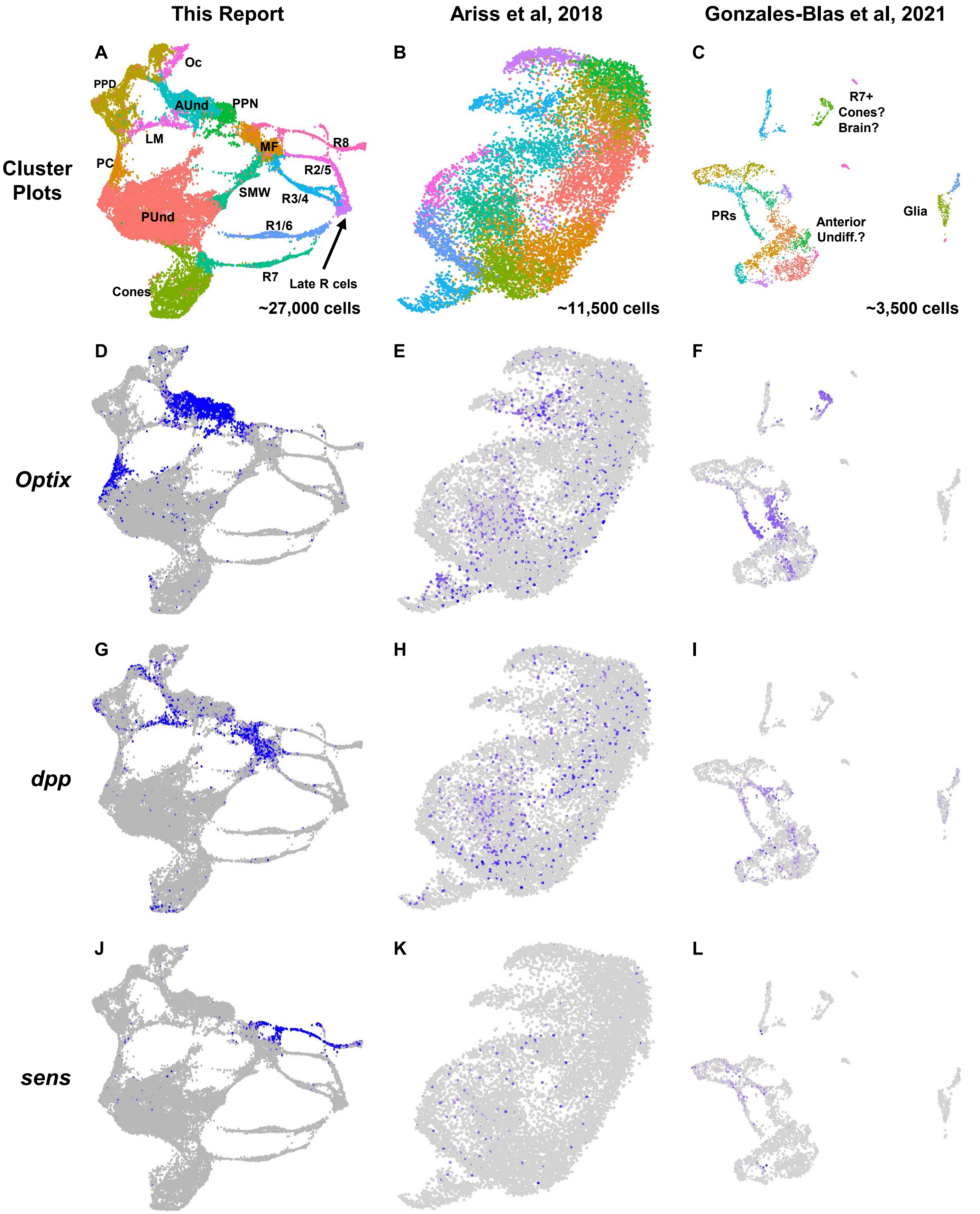

This Report
Ariss et al, 2018
Gonzales-Blas et al, 2021
A
B
C
Cluster Plots
Oc
PPD
AUnd
PPN
LM
MF
R8
PC
R2/5
SMW
R3/4
PUnd
R1/6
R7
Late R cels
Cones
~27,000 cells
~11,500 cells
R7+
Cones?
Brain?
PRs
Anterior
Undiff.?
Glia
~3,500 cells
D
E
F
Optix
G
H
I
dpp
J
K
L
sens

Supplementary Fig. 15: Comparison of scRNA-seq data sets generated from *Drosophila* larval eye discs. A. UMAP cluster plot of eye disc scRNA-seq data presented in this report showing ~27,000 cells with all expected cell identities. The data was generated using the 10x Genomics platform. **B.** UMAP cluster plot of ~11,500 cells from eye-antennal discs generated using Drop-seq technology. No cell subtype identities are readily identified. **C.** ~3,500 eye-antennal cells profiled using the 10x Genomics platform. Cell types are not well separated or represented. **D-L.** FeaturePlots showing *Optix* (**D-E**)*, dpp* (**G-I**) and *sens* (**J-L**) expression and distribution in UMAP plots shown in **A-C**. Most marker genes examined from the published data sets do not show cluster-specific expression.
