# Supplementary material for: A single cell genomics atlas of the Drosophila larval eye reveals distinct photoreceptor developmental timelines
Source: Nat Commun. 2023 Nov 8;14:7205. doi: 10.1038/s41467-023-43037-0 (PMC10632452; doi:10.1038/s41467-023-43037-0)
Supplement: Supplementary file 3 — Description of Additional Supplementary Files [file 41467_2023_43037_MOESM3_ESM.pdf]

Description of Additional Supplementary Files

Supplementary Data 1. Seurat called marker genes for each cell identity in scRNA-seq data.

Supplementary Data 2. Loading weight of each gene in the first principal component of all cell clusters in scRNA-seq data

Supplementary Data 3. Eigen values of each principal component for all cell clusters in scRNA-seq data

Supplementary Data 4. Differential accessible peaks for each cell identity in snATAC-seq data

Supplementary Data 5. Source data for Gene ontology term enrichment for photoreceptors and cones in snATAC-seq data
