# Supplementary material for: A single cell genomics atlas of the Drosophila larval eye reveals distinct photoreceptor developmental timelines
Source: Nat Commun. 2023 Nov 8;14:7205. doi: 10.1038/s41467-023-43037-0 (PMC10632452; doi:10.1038/s41467-023-43037-0)
Supplement: Supplementary file 9 — Reporting Summary [file 41467_2023_43037_MOESM9_ESM.pdf]

nature portfolio

Corresponding author(s): Graeme Mardon

Last updated by author(s): Sep 26, 2023

Reporting Summary

Nature Portfolio wishes to improve the reproducibility of the work that we publish. This form provides structure for consistency and transparency in reporting. For further information on Nature Portfolio policies, see our Editorial Policies and the Editorial Policy Checklist.

Statistics

For all statistical analyses, confirm that the following items are present in the figure legend, table legend, main text, or Methods section.

n/a	Confirmed	
☐	☒	The exact sample size (*n*) for each experimental group/condition, given as a discrete number and unit of measurement
☐	☒	A statement on whether measurements were taken from distinct samples or whether the same sample was measured repeatedly
☐	☒	The statistical test(s) used AND whether they are one- or two-sided<br>*Only common tests should be described solely by name; describe more complex techniques in the Methods section.*
☐	☐	A description of all covariates tested
☒	☐	A description of any assumptions or corrections, such as tests of normality and adjustment for multiple comparisons
☐	☒	A full description of the statistical parameters including central tendency (e.g. means) or other basic estimates (e.g. regression coefficient) AND variation (e.g. standard deviation) or associated estimates of uncertainty (e.g. confidence intervals)
☐	☒	For null hypothesis testing, the test statistic (e.g. *F*, *t*, *r*) with confidence intervals, effect sizes, degrees of freedom and *P* value noted<br>*Give P values as exact values whenever suitable.*
☒	☐	For Bayesian analysis, information on the choice of priors and Markov chain Monte Carlo settings
☒	☐	For hierarchical and complex designs, identification of the appropriate level for tests and full reporting of outcomes
☒	☐	Estimates of effect sizes (e.g. Cohen's *d*, Pearson's *r*), indicating how they were calculated

Our web collection on statistics for biologists contains articles on many of the points above.

Software and code

Policy information about availability of computer code

Data collection	Single cell libraries were generated using Chromium Next GEM Single Cell 3' Reagent Kit v3.1 from 10X Genomics for RNA and 10x Chromium Next GEM Single Cell ATAC kit v2 for ATAC experiments
Data analysis	We used Cellranger scRNA (cellranger 6.0.1) and scATAC (cellranger ATAC2.0) softwares fror initial analyses and of our data. Seurat (version4.03) and gSignac (version1.10)g was used for downstream analyses. Monocle3 software was used for pseudotime analyses. Panther 18.0 was used for GO analyses<br><br>All custom code is uploaded here: https://github.com/komalbollepogu/Drosophila_LarvalEye_SingleCell

For manuscripts utilizing custom algorithms or software that are central to the research but not yet described in published literature, software must be made available to editors and reviewers. We strongly encourage code deposition in a community repository (e.g. GitHub). See the Nature Portfolio guidelines for submitting code & software for further information.

Data

Policy information about availability of data

All manuscripts must include a data availability statement. This statement should provide the following information, where applicable:

- Accession codes, unique identifiers, or web links for publicly available datasets
- A description of any restrictions on data availability
- For clinical datasets or third party data, please ensure that the statement adheres to our policy

The raw and processed data generated in this study have been deposited in Gene Expression Omnibus under Accession number: GSE235110. Panther 18.0 database was used for GO analyses. The data availability statement is included in the manuscript. The Drosophila melanogaster reference genome Release 6 (dm6) was used

Research involving human participants, their data, or biological material

Policy information about studies with human participants or human data. See also policy information about sex, gender (identity/presentation), and sexual orientation and race, ethnicity and racism.

Reporting on sex and gender	N/A
Reporting on race, ethnicity, or other socially relevant groupings	N/A
Population characteristics	N/A
Recruitment	N/A
Ethics oversight	N/A

Note that full information on the approval of the study protocol must also be provided in the manuscript.

Field-specific reporting

Please select the one below that is the best fit for your research. If you are not sure, read the appropriate sections before making your selection.

☒ Life sciences ☐ Behavioural & social sciences ☐ Ecological, evolutionary & environmental sciences

For a reference copy of the document with all sections, see nature.com/documents/nr-reporting-summary-flat.pdf

Life sciences study design

All studies must disclose on these points even when the disclosure is negative.

Sample size	We have aabout 26,000 cells for scRNA seq experiments and ~20,000 cells for snATAC-seq experiments. One eye disc has 10,000 cells and therefore we have 2x coverage. 1x coverage is considered adequate for single cell experiments.
Data exclusions	No data was excluded
Replication	Replication is successful. The dissociation experiments were performed several times over a period of one year successfully. The scRNA-seq data was generated by 2 biological replicates that were performed 2 months apart successfully. The snATAC-seq data was successfully generated few months apart from 3 independent biological repeats
Randomization	We performed experiments on Drosophila male eye disc. It is expected that males and females do not show transcriptomic difference in the eye. We used males to capture genes on the Y chromosome
Blinding	Not applicable

Reporting for specific materials, systems and methods

We require information from authors about some types of materials, experimental systems and methods used in many studies. Here, indicate whether each material, system or method listed is relevant to your study. If you are not sure if a list item applies to your research, read the appropriate section before selecting a response.

Materials & experimental systems

n/a	Involved in the study
☐	☒ Antibodies
☒	☐ Eukaryotic cell lines
☒	☐ Palaeontology and archaeology
☐	☒ Animals and other organisms
☒	☐ Clinical data
☒	☐ Dual use research of concern
☒	☐ Plants

Methods

n/a	Involved in the study
☒	☐ ChIP-seq
☒	☐ Flow cytometry
☒	☐ MRI-based neuroimaging

Antibodies

Antibodies used	We used the following antibodies: rat anti-Elav from DHSB-7E8A10(RRID:AB, #52818, 1:500), chicken anti-GFP (Abcam. Catalog Number: ab13970, RRID:AB, #300798, 1:1000), rabbit anti-mCherry (Thermofischer scientific, Catalog # MA5-47061,RRID:AB, #2889995, 1:2000), guinea pig anti-Runt (gift from Dr. Claude Desplan), guinea pig anti-Sens (a gift from Hugo Bellen, 1:1000) and mouse anti-Svp from DHSB-2D3 (RRID:AB, #2618079, 1:500). The following secondary antibodies were used at 1:500 concentration: Cy5 anti-rat (Jackson Immunoresearch, catalog # 712-175-153,RRID: AB, #2534067), Cy5 anti-guinea pig (Abcam, Catalog # ab102372,RRID:AB, #2340460), Alexa 488 anti-guinea pig (Thermofischer Scientific, Catalog # A-11073,RRID: AB, #2534117), Alexa 488 anti-chicken (Thermofischer Scientific, Catalog # A-11039,RRID:AB, #2762843), Alexa 568 anti-rabbit (Thermofischer Scientific,Catalog # A10042,RRID:AB, #2534017), Alexa 488 anti-mouse (Thermofischer Scientific, Catalog # A-11029,RRID: AB, #2536161) and Alexa 555 anti-rat (Thermofischer Scientific,Catalog # A-21434,RRID: AB, #2535855).
Validation	All antibodies have been previously validated and most of them are available on DHSB

Animals and other research organisms

Policy information about studies involving animals; ARRIVE guidelines recommended for reporting animal research, and Sex and Gender in Research

Laboratory animals	Drosophila melanogaster cantons male late larval eye discs. Age is 0 hour after egg laying
Wild animals	No wild animals ere included
Reporting on sex	We performed experiments on Drosophila male eye disc. It is expected that males and females do not show transcriptomic difference in the eye. We used males to capture genes on the Y chromosome
Field-collected samples	No field samples were used
Ethics oversight	No ethical guidance required as the study does not involve human subjects or harmful substances

Note that full information on the approval of the study protocol must also be provided in the manuscript.
